# Tentative identification of gefitinib metabolites in non-small-cell lung cancer patient plasma using ultra-performance liquid chromatography coupled with triple quadrupole time-of-flight mass spectrometry

**DOI:** 10.1371/journal.pone.0236523

**Published:** 2020-07-23

**Authors:** Chao Wang, Jingui Zhang, Simin Zhou, Limei Yu, Fangxuan Han, Ren Ling, Jin Ling

**Affiliations:** 1 Department of Pharmacy, Hainan General Hospital, Hainan Affiliated Hospital of Hainan Medical University, Haikou, Hainan, China; 2 Phase I Clinical Trial Ward/Department of Pharmacy, Hainan General Hospital, Hainan Affiliated Hospital of Hainan Medical University, Haikou, Hainan, China; 3 Department of Function Examination, the Children’s Hospital of Jiangxi Province, Nanchang, Jiangxi, China; 4 Department of Pathology, Zhejiang Jinhua Guangfu Hospital, Jinhua, Zhejiang, China; 5 Department of Pharmacy, Zhejiang Jinhua Guangfu Hospital, Jinhua, Zhejiang, China; 6 Department of Pharmaceutical Analysis, School of Pharmacy, Fudan University, Shanghai, China; Fisheries and Oceans Canada, CANADA

## Abstract

**Background:**

Gefitinib is an orally potent and selective ATP-competitive inhibitor of epidermal growth factor receptor (EGFR) tyrosine kinase and is commonly used to treat locally advanced or metastatic non-small-cell lung cancer (NSCLC) with sensitive EGFR mutations. Multiple adverse effects associated with gefitinib, including liver and lung injuries, severe nausea, and diarrhea, have limited its clinical application. Xenobiotic-induced bioactivation is thought to be an important reason for gefitinib toxicity, which encouraged us to clarify the metabolism of gefitinib in NSCLC patients.

**Materials and methods:**

An ultra-performance liquid chromatography coupled with triple quadrupole time-of-flight mass spectrometry (UPLCQ-TOF-MS) method was established to tentatively identify the metabolites of gefitinib in human plasma. The extracted ion chromatogram peak intensity threshold was set at 1500 cps with minimum MS and MS/MS peak intensities of 400 and 100 cps, respectively.

**Results:**

A total of 18 tentative metabolites were identified. Eight novel tentative metabolites with metabolic changes in dechlorination, defluorination, and hydrogenation on the quinazoline skeleton; removal of a partial or complete 3-chloro-4-fluoroaniline-substituted group; and sulfate conjugation and taurine conjugation were newly discovered in human plasma. Based on structural analysis of the tentative metabolites, the metabolic pathways were proposed. In addition, the pathways of dechlorination, defluorination, and hydrogenation on the quinazoline skeleton; removal of partial or complete 3-chloro-4-fluoroaniline-substituted groups; and sulfate conjugation and taurine conjugation in humans *in vivo* indicate that novel metabolic pathways exist in humans.

**Conclusions:**

In summary, the metabolism of gefitinib in humans *in vivo* is extensive and complex. Based on *in vivo* evidence, the propoxy-morpholine ring side chain and O-methyl group are the critical metabolic regions of gefitinib in humans. The novel metabolic pathways differ from those of *in vitro* studies, suggesting that intestinal floral metabolism might be involved.

## Introduction

Epidermal growth factor receptor (EGRF) tyrosine kinase plays a fundamental role in the RAS-RAF-ErK, PI3K-AKT/PKC-NFκB and Ral-c-Src-STAT signal pathways [1–3]. Cellular transformation is enhanced with mutation or overexpression of EGFR as well as erbB2, leading to a poor prognosis in cancer patients, especially those with non-small-cell lung cancer (NSCLC) [4, 5]. Gefitinib (Iressa), an orally potent and selective ATP-competitive inhibitor of EGFR tyrosine kinase, is commonly used to treat locally advanced or metastatic NSCLC with sensitive EGFR mutations [6]. The progression-free survival and overall survival of NSCLC patients with EGFR-active gene mutations are significantly improved after treatment with gefitinib, and as a result, the FDA approved gefitinib as a first-line therapy for NSCLC in 2015 [7, 8]. However, multiple adverse effects associated with gefitinib, such as liver and lung injuries, severe nausea, and diarrhea, have limited its clinical application. Investigation of gefitinib metabolism in humans could help to optimize its pharmacokinetics and avoid drug toxicity [3, 6, 9]. Additionally, discovery of active metabolites might lead to new or improved drug candidates.

Gefitinib is a small-molecule compound with a chemical structure of 4-quinazolinamine, N-(4-fluorophenyl)-7-methoxy-6-[3-(4-morpholinyl) propoxy] [6]. In a previous study, metabolites of gefitinib were investigated using a target metabolite identification strategy. [^14^C]-labeled gefitinib and potential metabolite compounds were synthesized and analyzed using LC-radiochemical-mass spectrometry. In an *in vitro* study, gefitinib was confirmed to be metabolized extensively by cytochrome P-450 enzymes [10, 11]. Gefitinib was metabolized primarily by CPY3A4 and to a lesser extent by CYP2D6 and 3A5, producing a series of metabolites to human liver microsomes. The main metabolic routes of gefitinib in human liver microsomes were oxidation of the morpholine ring, oxidative defluorination and O-demethylation of the methoxy-substituent on a quinazoline nucleus. In the *in vivo* study, the metabolites of gefitinib in rat and dog models and in healthy human volunteers were investigated after oral administration of isotopically labeled gefitinib [12, 13]. In rats, the major route of metabolism was morpholine ring oxidation, and M537194 and M608236 were the primary metabolites in rat plasma. In dogs, metabolism of the N-propoxy-morpholine group, O-demethylation of the methoxy-substituent on the quinazoline ring structure and oxidative defluorination of the halogenated phenyl group were observed. In a previous *in vivo* human study, O-demethylation and morpholine ring oxidation were identified as the major routes of metabolism [14] (S1 Fig).

Excellent metabolite detection performance is achieved using ultra-performance liquid chromatography coupled with triple quadrupole time-of-flight mass tandem mass spectrometry (UPLC-Q-TOF-MS/MS) with high detection speed and sensitivity [15]. Full-scan MS and MS/MS spectra data sets for each potential metabolite can be obtained in a single run. Q-TOF-MS/MS not only supplies accurate masses of chemicals but also offers valuable and informative MS/MS spectra. Analysis of the data sets enables identification of both target and nontarget metabolites. In target metabolite identification research, as in the previous study mentioned above, all potential metabolites are synthesized and analyzed to verify whether they exist in the samples as metabolites [10, 12, 16]. Limit metabolites are found following the target metabolite identification strategy. However, nontarget metabolite analysis using UPLC-Q-TOF-MS/MS can supply a variety of potential metabolites, which can aid in discovery of unknown metabolites.

To date, no *in vivo* study of gefitinib metabolism has been conducted in NSCLC patients. An investigation that explores the relationships among the dosage regimen, the exposure of gefitinib and its metabolites, and the drug effects in NSCLC patients is ongoing at our institute, and hence, a detection method is warranted to simultaneously analyze the exposures of gefitinib and its main metabolites. In this study, plasma samples obtained from NSCLC patients after oral administration of gefitinib were analyzed using UPLC-Q-TOF-MS/MS, and 18 tentative metabolites were tentatively identified based on accurate mass measurements and fragmentation patterns. The metabolic pathways were also proposed.

## Materials and methods

### Chemicals

The reference standards of gefitinib (Product number: Y0001809, Lot number: BCCB6739), MS-grade acetonitrile (Product number: 1.00029.2500, Lot number: I1066929001), and formic acid (Product number: 94318-250ML-F, Lot number: BCBN7383V) for UPLC-Q-TOF-MS/MS analysis were purchased from Merck Company (Darmstadt, Germany). Deionized water was freshly processed through a Milli-Q water purification system (Millipore, USA).

### Clinical study design and sample collection

Inclusion criteria: All patients are pathologically diagnosed as NSCLC, cases have complete clinical information, patients must be aged from more than 18 years to less than 60 years at the time of study enrollment. Patient who has a severe and uncontrollable medical disease, has a chronic liver disease, uncontrolled intercurrent illness has to be excluded. Finally, three NSCLC patients in Zhejiang Jinhua Guangfu hospital treated with gefitinib were involved following good clinical practice guidelines and the guiding principles of the Declaration of Helsinki and the participant recruitment date period was from 16th August, 2019 to 17th September, 2019. All three patients are male and aged from 47 to 54 years. Each patient signed an informed consent form, and the protocol was approved by the ethics committee of Zhejiang Jinhua Guangfu hospital and authors had access to information that could identify individual participants during and after data collection. Blood samples were collected into heparin vacuum tubes before and after one week of once-a-day oral administration of 250 mg gefitinib. The blood samples were centrifuged at 3,000 g for 5 minutes, and the plasma samples were collected and stored at -80°C before analysis.

### Sample preparation

The plasma samples were thawed at 4°C prior for analysis. Six hundred microliters of acetonitrile was added to 200 μL of plasma sample and vortex mixed for 3 minutes. The samples were centrifuged at 10,000 g for 10 minutes to precipitate proteins. After centrifugation, the supernatant was filtered through a 0.22 μm membrane and transferred to another tube. An aliquot of 10 μL of supernatant was injected for UPLC-Q-TOF-MS/MS analysis.

### UPLC-Q-TOF-MS/MS conditions

Analysis of gefitinib and metabolites was performed on an Agilent 1290 UPLC system coupled to an AB Sciex TripleTOF 5600+ system (a hybrid quadrupole time of flight tandem mass spectrometer equipped with Turbo V sources and a Turbo ion spray interface). An Eclipse plus C18 reverse-phase LC column (2.1 x 50 mm i.d.; 1.8 μm, Agilent, USA) was selected for chromatographic analysis. The column temperature was set as 40°C. The mobile phase consisted of solvent A (0.5% formic acid aqueous solution, v/v) and solvent B (acetonitrile) with a flow rate of 0.2 mL/min. The gradient elution program was optimized as follows: 0–1.00 minute (5–5% B), 1.01–1.50 minutes (5–15% B), 1.51–6.00 minutes (15–25% B), 6.01–12.00 minutes (25–65% B), 12.01–13.00 minutes (65% B), and 13.01–15.00 minutes (65–5% B) [17–20]. The sample injection volume was 10 μL. The mass spectrometer was equipped with an ESI ion source operating in positive ion mode. The conditions of the MS/MS detector are listed as follows: source temperature = 180°C, ion spray voltage = 5.0 kV, declustering potential (DP) = 80 V and collision energy (CE) = 40 eV. The fragmentation patterns of the precursor ion were obtained under collision-induced dissociation (CID) mode. Nitrogen was selected as the nebulizer gas (Gas 1), heater gas (Gas 2) and curtain gas, the parameters of which were set at 50, 50 and 30 psi, respectively. The mass range and accumulation time in positive full-scan mode were set to 100 to 1000 amu and 0.25 sec, respectively. The six most intense precursor ions were selected for an MS/MS scan. Fragment ions ranging from 50 to 1000 amu of each precursor ion were detected and exceeded 50 cps counts for further production scanning were performed within a 0.1 sec accumulation time. Raw data were acquired by using Analyst^®^ TF 1.6 software (AB Sciex).

### Data analysis

The retention time and mass spectra of a series of extracted ion chromatograms (XICs) of the experimental samples were compared with those of blank samples using the MasterView^®^ program (v1.1, AB Sciex) to discover potential metabolites. The extracted ion chromatogram peak intensity threshold was set at 1500 cps with minimum MS and MS/MS peak intensities of 400 and 100 cps, respectively. The structures of potential gefitinib metabolites were determined by considering the mass shift, fragmentation patterns, and retention time. The assigned metabolite structures were further matched with the MS/MS spectra using MetabolitePilot^™^ software (v2.0, AB Sciex).

## Results

### Structural characterization of gefitinib

**Fig 1:**
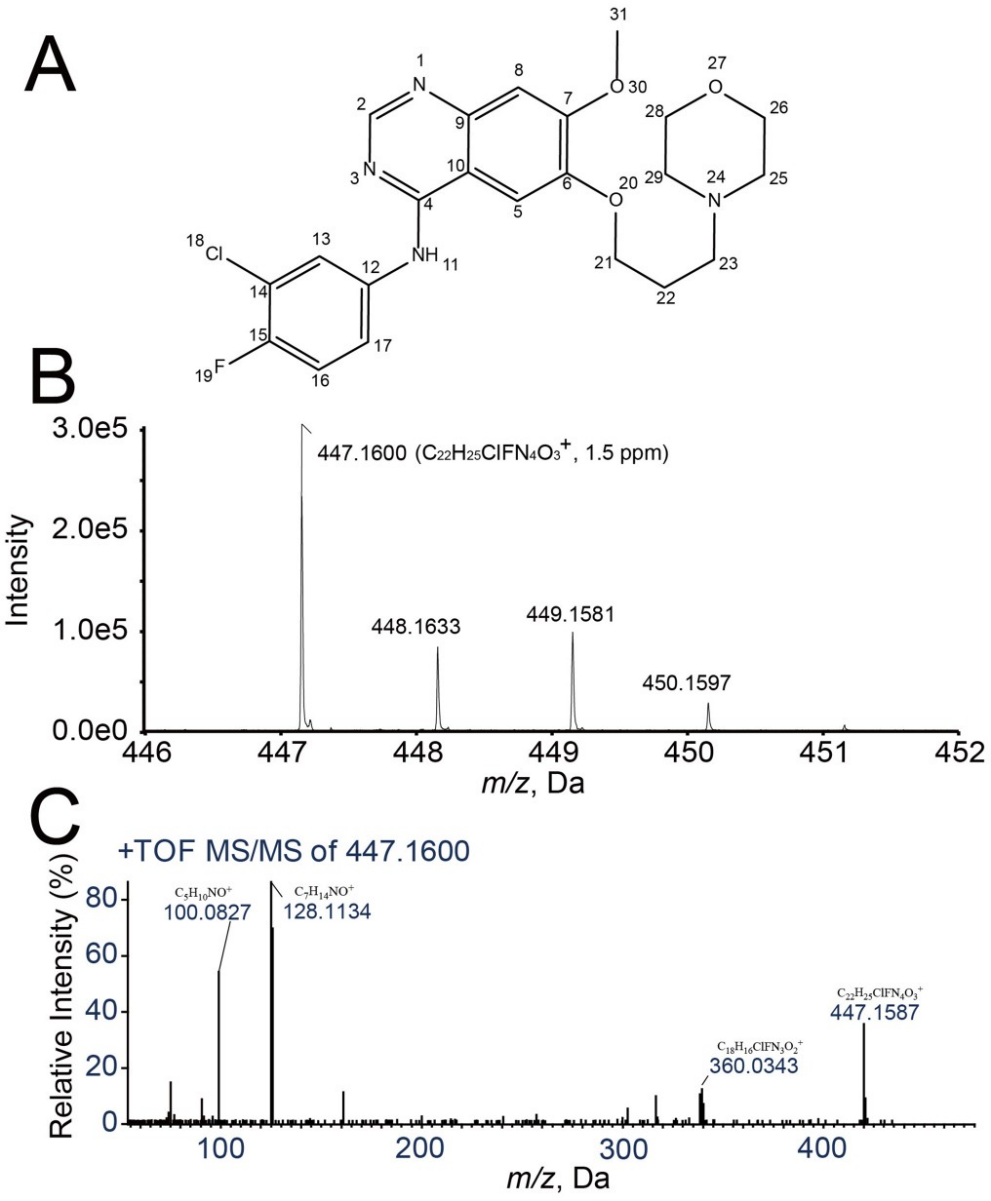
(A) Chemical structure of gefitinib and assigned atomic numbers. (B) Full-scan mass spectrum of gefitinib. (C) MS/MS spectrum at *m/z* 447.1600 and fragment pattern assignment of gefitinib.

**Fig 2:**
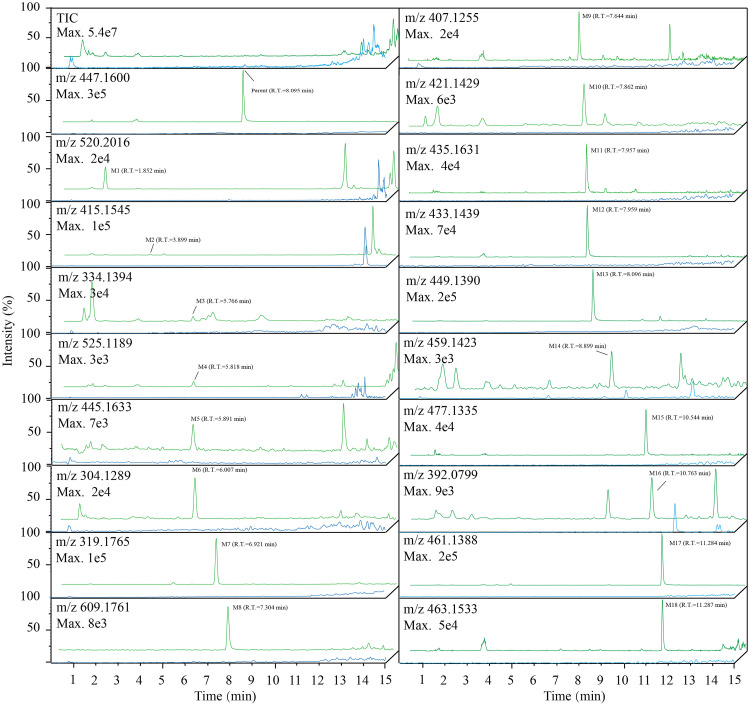
Full scan chromatograms and extracted ion chromatograms [M+H]^+^ of gefitinib and its tentative metabolites in human plasma. Line chart in green: Sample group. Plasma collected after oral administration of gefitinib. Line chart in blue: Control group. Plasma collected before oral administration of gefitinib. The parent drug and 18 tentative metabolites were discovered by comparing sample group with control group and are labeled in order of retention time.

The protonated ion of gefitinib with *m/z* 447.1546 (mass accuracy: -1.6 ppm) was observed, and the retention time of the target peak was 8.095 minutes (Figs 1 and 2). The MS/MS spectrum of the gefitinib reference standard showed product ions with *m/z* 100.0827, 128.1134 and 360.0343. The prominent product ions with *m/z* 128.1134 and 100.0827 were assigned to the propoxy-morpholine ring side chain and the morpholine ring on the side chain, respectively. The remaining product ions with *m/z* 360.0343 was assigned to the parent compound with loss of morpholine ring (Fig 1).

### Tentative identification of gefitinib metabolites

Fig 2 shows the full scan chromatograms and chromatograms of the extracted ion of tentative metabolites in the NSCLC patient plasma before or after treatment with oral administration of gefitinib. Eighteen tentative metabolites were discovered by comparing the samples with the blanks and were numbered consecutively according to their chromatographic retention time. Structural characterization of the tentative metabolite was processed based on the exact molecular mass from the full scan, the MS/MS spectrum and the proposed metabolic pathways (summarized in Table 1). The details of structure analysis were shown in S1 File.

**Table 1:** Summary of tentative identification of gefitinib metabolites in human plasma.

Tentative metabolite	Retention time (min)	Measured *m/z* (M+H)	Predicted molecular formula	Mass error (ppm)	Identification	Fragment ions (Integer values)
Gefitinib	8.095	447.1600	C_22_H_24_ClFN_4_O_3_	1.5	GEF	100, 128, 271, 317, 360
M1	1.852	520.2016	C_24_H_30_FN_5_O_5_S	-1.6	GEF-Cl+Taurine	120, 166, 190, 208, 355
M2	3.899	415.1545	C_21_H_22_ClN_4_O_3_	4.3	GEF-F-Methyl	-
M3	5.766	334.1394	C_16_H_19_N_3_O_5_	-1.0	GEF-C6H3NClF-2H+O	118, 146, 188
M4	5.818	525.1189	C_22_H_26_ClN_4_O_7_S	-2.5	GEF-F+O+Sulfate	100, 128, 445
M5	5.891	445.1633	C_22_H_25_ClN_4_O_4_	-0.9	GEF-F+O	100, 128, 341
M6	6.007	304.1289	C_16_H_22_N_3_O_3_	-2.1	GEF-C6H3NClF	100, 128, 145, 245
M7	6.921	319.1765	C_16_H_22_N_4_O_3_	-1.7	GEF-C6H2ClF	100, 128, 260, 319
M8	7.304	609.1761	C_27_H_30_ClFN_4_O_9_	-0.4	GEF-Methyl+Glucuronide	128, 346, 433
M9	7.644	407.1255	C_19_H_20_ClFN_4_O_3_	-1.8	GEF-3 Methyl+2H	74, 102, 306, 346
M10	7.862	421.1429	C_20_H_22_ClFN_4_O_3_	2.9	GEF-2Methyl+2H	74, 102, 305, 320
M11	7.957	435.1631	C_21_H_24_ClFN_4_O_3_	1.4	GEF-Methyl+2H	100, 128, 306, 320, 348
M12	7.959	433.1439	C_21_H_22_ClFN_4_O_3_	0.5	GEF-Methyl	100, 128, 346
M13	8.096	449.1390	C_21_H_22_ClFN_4_O_4_	-0.2	GEF-Methyl+O	74, 128, 376
M14	8.899	459.1423	C_22_H_23_ClN_4_O_5_	-1.4	GEF-F+2O-2H	86, 142, 318
M15	10.544	477.1335	C_22_H_22_ClFN_4_O_5_	-0.1	GEF+2O-2H	158, 304, 320
M16	10.763	392.0799	C_18_H_15_ClFN_3_O_4_	-2.4	GEF-Morpholine+2O-2H	-
M17	11.284	461.1388	C_22_H_22_ClFN_4_O_5_	0.4	GEF+O-2H	86, 142, 320
M18	11.287	463.1533	C_22_H_24_ClFN_4_O_5_	0.5	GEF+O-2H+2H	86, 114, 142, 322

**Fig 3:**
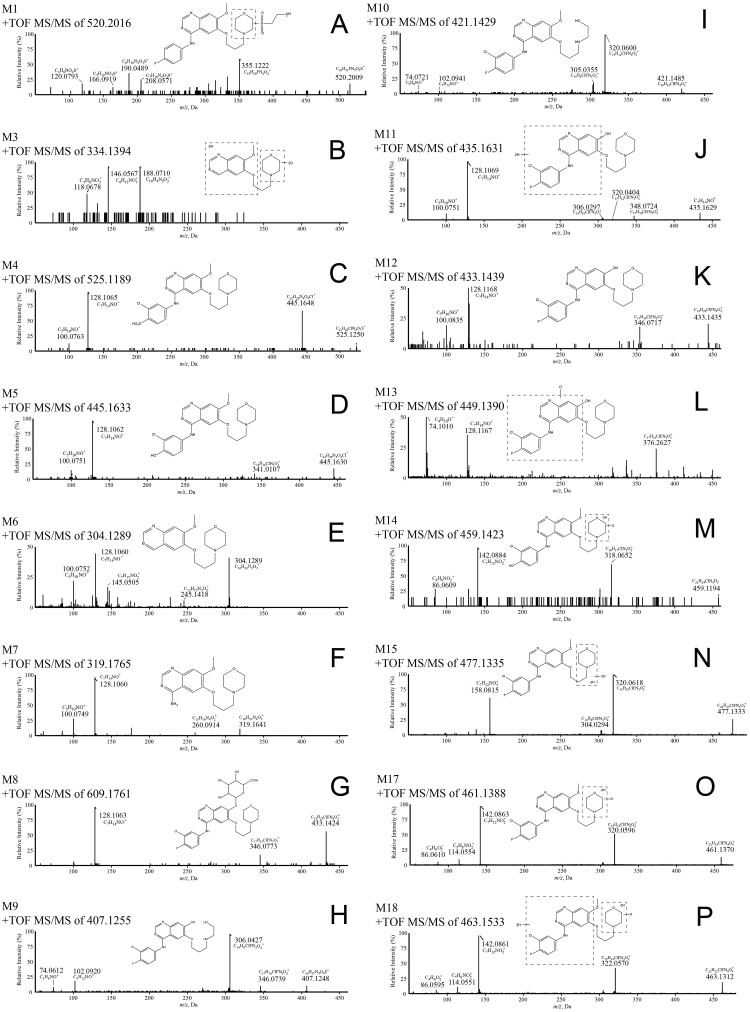
MS/MS spectra of sixteen tentative metabolites obtained from their respective precursors under collision-induced dissociation. The structures were elucidated by analyzing the fragmentation patterns and confirmed using structure matching software MetabolitePilot^™^.

Tentative metabolite M1: The tentative metabolite M1 was detected at 1.852 minutes. The accurate mass of the protonated ion of M1 was 520.2016 with a proposed molecular formula of C_24_H_30_FN_5_O_5_S (mass accuracy: -1.6 ppm). Replacement of a chlorine atom with a hydrogen atom and addition of taurine (-34+107 Da from the parent compound) were suggested with the molecular formula. The proposed structure was shown in Fig 3A.

Tentative metabolite M2: The retention time of M2 was 3.899 minutes. The accurate mass of the protonated ion of M2 was 415.1545, and the molecular formula of M2 was proposed to C_21_H_22_ClN_4_O_3_(mass accuracy: 4.3 ppm). Because the MS/MS spectrum of M2 was not obtained, M2 was considered a compound by metabolizing gefitinib with defluorination at the F-19 position and demethylation at the O-30 position (-18-14 Da from the parent compound) based on the measured exact molecular mass and the most likely metabolic position of parent compound structure.

Tentative metabolite M3: The retention time of M3 was 5.766 minutes. The tentative metabolite M3 was detected with a protonated ion of 334.1394, and the molecular formula of M3 was proposed to be C_16_H_19_N_3_O_5_ (mass accuracy: -1.0 ppm). The molecular formula of M3 suggested removal of the 3-chloro-4-fluoroaniline substitutional group, addition of two oxygen atoms and dehydrogenation (-143+32–2 Da from the parent compound). The proposed structure was shown in Fig 3B.

Tentative metabolite M4: The retention time of M4 was 5.818 minutes. The accurate mass of the protonated ion of M4 was 525.1189, and the molecular formula of M4 was proposed to be C_22_H_26_ClN_4_O_7_S (mass accuracy: -2.5 ppm). The molecular formula of M4 indicated loss of the fluorine atom and addition of a sulfate group (-18+96 Da from the parent compound). The proposed structure was shown in Fig 3C.

Tentative metabolite M5: The retention time of M5 was 5.891 minutes, and the accurate mass of the protonated ion of M5 was 445.1633. The proposed molecular formula C_22_H_25_ClN_4_O_4_ of M5 (mass accuracy: -0.9 ppm) suggested defluorination and hydroxylation of gefitinib (-18+16 Da from the parent compound). The proposed structure was shown in Fig 3D.

Tentative metabolite M6: The retention time of M6 was 6.007 minutes. The molecular formula of M6 was proposed to be C_16_H_22_N_3_O_3_ (mass accuracy: -2.1 ppm) based on the protonated ion of *m/z* 304.1289. The molecular formula of M6 suggested that the 3-chloro-4-fluoroaniline substitutional group on gefitinib was removed on M6 (-143 Da from the parent compound). The proposed structure was shown in Fig 3E.

Tentative metabolite M7: The tentative metabolite M7 was detected at 6.921 minutes. The accurate mass of the protonated ion of M7 was 319.1765, and the molecular formula of M7 was proposed to be C_16_H_22_N_4_O_3_ (mass accuracy: -1.7 ppm). The molecular formula of M7 indicated loss of the 1-chloro-2-fluorobenzene group from gefitinib (-128 Da from the parent compound). The proposed structure was shown in Fig 3F.

Tentative metabolite M8: The retention time of M8 was 7.304 minutes, and the accurate mass of the protonated ion of M8 was 609.1761. The molecular formula C_27_H_30_ClFN_4_O_9_ (mass accuracy: -0.4 ppm) of M8 suggested that the parent drug was metabolized by demethylation and glucuronide conjugation (-14+176 Da from the parent compound). The proposed structure was shown in Fig 3G.

Tentative metabolite M9: The tentative metabolite M9 was detected at 7.644 minutes. The accurate mass of the protonated ion of M9 was 407.1255, and the molecular formula was proposed to be C_19_H_20_ClFN_4_O_3_ (mass accuracy: -1.8 ppm). The molecular formula of M9 suggested triple demethylation and hydrogenation of gefitinib (-42+2 Da from the parent compound). The proposed structure was shown in Fig 3H.

Tentative metabolite M10: The retention time of M10 was 7.862 minutes. The accurate mass of the protonated ion of M10 was 421.1429. The molecular formula of M10 was proposed to be C_20_H_22_ClFN_4_O_3_ (mass accuracy: 2.9 ppm). The molecular formula of M10 suggested that the parent drug was metabolized by double demethylation and hydrogenation (-28+2 Da from the parent compound). The proposed structure was shown in Fig 3I.

Tentative metabolite M11: The retention time of M11 was 7.957 minutes, and the accurate mass of the protonated ion of M11 was 435.1231. The proposed molecular formula of M11 C_21_H_24_ClFN_4_O_3_ (mass accuracy: 1.9 ppm) suggested that gefitinib was metabolized by demethylation and hydrogenation (-14+2 Da from the parent compound). The proposed structure was shown in Fig 3J.

Tentative metabolite M12: The retention time of M12 was detected at 7.959 minutes. The accurate mass of the protonated ion of M11 was 433.1439, and the molecular formula of M12 was proposed to be C_21_H_24_ClFN_4_O_3_ (mass accuracy: 0.5 ppm). The molecular formula of M12 suggested that the parent drug was metabolized by demethylation (-14 Da from the parent compound). The proposed structure was shown in Fig 3K.

Tentative metabolite M13: The retention time of M13 was 8.096 minutes. The accurate mass of the protonated ion of M13 was 449.1390, and the molecular formula of M13 was proposed to be C_21_H_22_ClFN_4_O_4_ (mass accuracy: -0.2 ppm). The molecular formula of M13 suggested that gefitinib was metabolized by demethylation and oxidation (-14+16 Da from the parent compound). The proposed structure was shown in Fig 3L.

Tentative metabolite M14: The retention time of M14 was 8.899 minutes. The accurate mass of the protonated ion of M14 was 459.1423, and the molecular formula of M14 was proposed to be C_22_H_23_ClN_4_O_5_ (mass accuracy: -1.4 ppm). The molecular formula of M14 suggested that the parent drug was metabolized by defluorination, oxidation and dehydrogenation (-18+32–2 Da from the parent compound). The proposed structure was shown in Fig 3M.

Tentative metabolite M15: Double hydroxylation and dehydrogenation (+32–2 Da from the parent compound). The retention time of M15 was detected at 10.544 minutes, and the accurate mass of the protonated ion was 477.1335. The molecular formula of M15 proposed to be C_22_H_22_ClFN_4_O_5_ (mass accuracy: -0.1 ppm) suggested that the parent drug was metabolized by double hydroxylation and dehydrogenation. The proposed structure was shown in Fig 3N.

Tentative metabolite M16: The retention time of M16 was 10.763 minutes. The accurate mass of the protonated ion of M16 was 392.0799, and the molecular formula of M16 was proposed to be C_18_H_15_ClFN_3_O_4_ (mass accuracy: -2.4 ppm). The molecular formula of M16 suggested that the parent drug was metabolized by loss of morpholine, hydroxylation and carbonylation (-85+16+14 Da from the parent compound).

Tentative metabolite M17: The retention time of M17 was 11.284 minutes. The accurate mass of the protonated ion of M17 was 461.1388, and the molecular formula of M17 was proposed as C_22_H_22_ClFN_4_O_5_ (mass accuracy: 0.4 ppm). The molecular formula of M17 suggested that the parent drug was metabolized by oxidation and dehydrogenation. The proposed structure was shown in Fig 3O.

Tentative metabolite M18: The retention time of M18 were 11.287 minutes. The protonated ion of M18 had an accurate mass of 463.1533, approximately 2 Da more than that of M17, suggesting an addition of two hydrogen atoms from M17. Therefore, the molecular formula of M18 was proposed to be C_22_H_24_ClFN_4_O_5_ (mass accuracy: 0.5 ppm). M18 was identified as hydrogenated M17. The proposed structure was shown in Fig 3P.

### Tentative metabolites and metabolic pathways

The tentative metabolites were divided into phase I and phase II groups based on the metabolic pathway.

There were 16 tentative metabolites in the phase I group including M2~7 and M9~18. Structural modification with oxidation, dehydrogenation, hydrogenation, defluorination and demethylation were observed in phase I group. Tentative metabolites in the phase I group could be divided into three subgroups based on the positions of structural modification. **Subgroup 1**: Structural modification on propoxy-morpholine ring side chain. Tentative metabolites M3, M9, M14, M15, M17, and M18 were included. Tentative metabolites M14, M17 and M18 had oxidation and dehydrogenation modifications on the propoxy-morpholine ring side chain concurrently. Tentative metabolites M3 and M15 had a reoxidation modification on the propoxy-morpholine ring side chain. Tentative metabolite M9 had a ring-opening modification. **Subgroup 2**: Structural modification on quinazoline. Tentative metabolites M2, M9, M11, M12, M13 and M18 were included. Tentative metabolites M2, M9, M11, M12 and M13 had a demethylation modification at O-30 position. Tentative metabolites M13 and M18 had oxidation and hydrogenation modifications on quinazoline or N-[1-chloro-2-fluorobenzene] substituent group, respectively. **Subgroup 3**: Structural modification on N-[1-chloro-2-fluorobenzene] substituent group. Tentative metabolites M2, M3, M4, M5, M6, M7 and M14 were included. Tentative metabolites M2, M4, M5 M14 had defluorination or substituted modification at F-19 position. Tentative metabolites M3, M6 and M7 had a loss of N-[1-chloro-2-fluorobenzene] substituent group modification.

There were 2 tentative metabolites in the phase II group including M1 and M8. Tentative metabolite M1 had a taurine conjugation on the morpholine ring of side chain. Tentative metabolite M8 had a glucuronide conjugation at the O-30 position.

**Fig 4:**
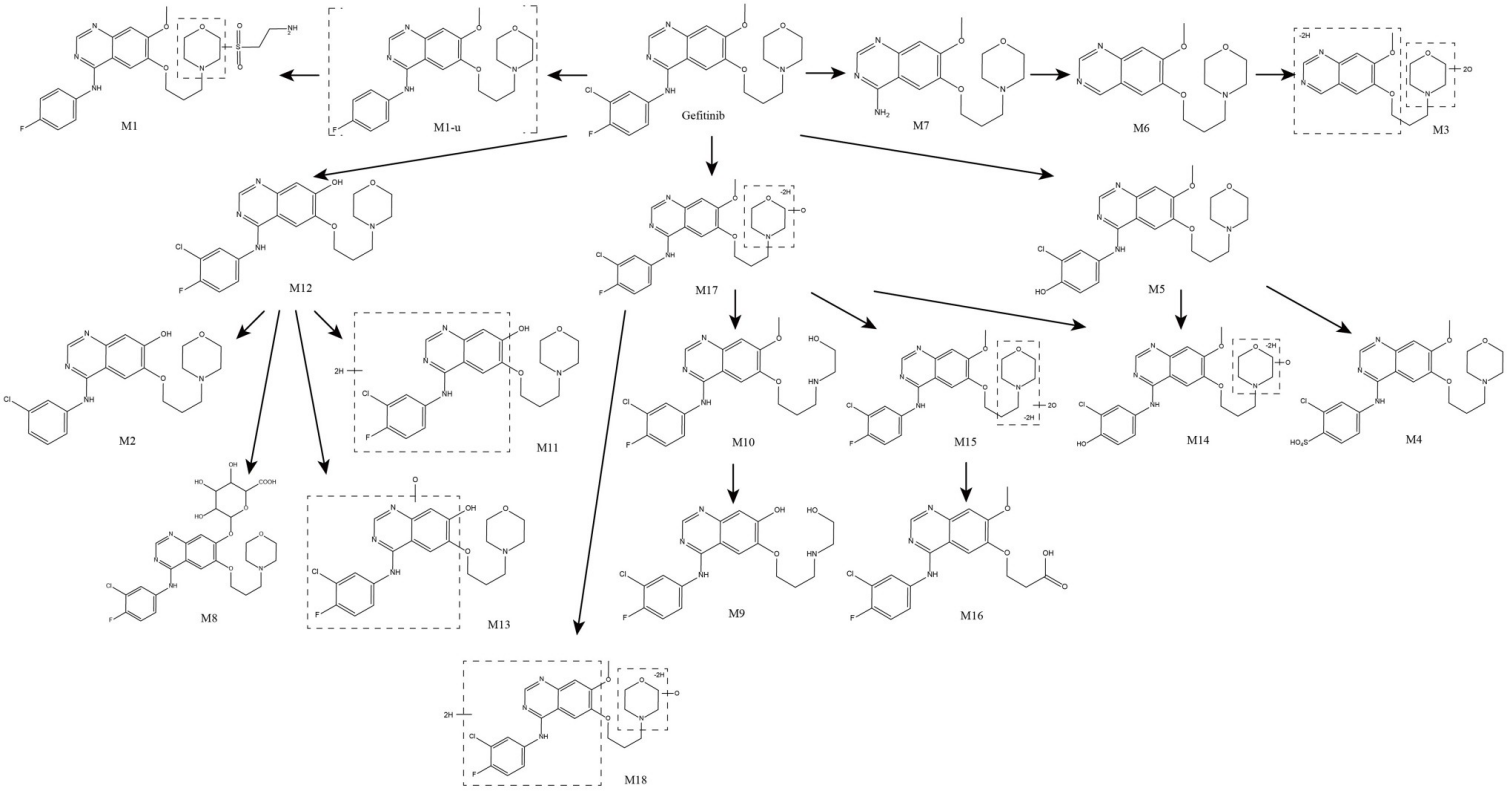
Proposed metabolic pathways of gefitinib in humans in vivo. Compound marked with dotted bracket is given to elucidate the proposed metabolic pathways, which were not observed.

The metabolic pathways of gefitinib in humans *in vivo* were proposed (Fig 4). Oxidation and dehydrogenation were the first step of metabolic changes in the morpholine ring, followed by ring-opening and partial or even complete removal of the morpholine ring, producing M9, M10 and M16. An addition of oxidation on the propoxy-morpholine ring chain after the first step of oxidation was also observed in M3, M14, M15 and M16. O-desmethyl gefitinib was further metabolized by defluorination, sulfate conjugation, hydrogenation and oxidation into M2, M8, M11 and M13, respectively. O-demethylation also occurred while M10 was transformed into M9. In addition, the fluorine atom substituted by a hydroxyl group or sulfate group was observed in M4, M5 and M14. Tentative metabolite M1 was produced by dechlorination and taurine conjugation against gefitinib. The gefitinib was proposed to be metabolized by dechlorination at the first step into M1-u, which was not detected, and further metabolized by taurine conjugation. Moreover, the removal of a partial or complete 3-chloro-4-fluoroaniline-substituted group was observed in M3, M6 and M7, which was proposed as another metabolic process.

## Discussion

In this study, an UPLC-TOF-MS/MS-based metabolite identification approach was employed to tentatively identified gefitinib metabolites and profile gefitinib metabolism in human. The results suggested that gefitinib was extensively metabolized in humans. The morpholine ring was the critical metabolic region of gefitinib because nine out of eighteen tentative metabolites with metabolic changes in the morpholine ring were identified. The oxidative degradation of the propoxy-morpholine side chain and this resulted in ring-opening, with partial and complete removal of the morpholine ring and propoxy side chain were observed in tentative metabolites M3, M9, M10, M14, M15, M16, M17 and M18. The extensive range of metabolites formed was similar to that found during metabolism of other compounds with chemical structure of morpholine ring, including timolol, moclobemide and the renin inhibitor, A-74273 [10]. In the previous study [11], CYP3A4 was demonstrated to be a major enzyme contributing to oxidation of the propoxy-morpholine ring side chain and oxidative defluorination. In this study, same metabolites were identified as that of the previous study. As a result, CYP3A4 was considered as a primary enzyme of gefitinib metabolism in human.

The second most important metabolic change was suggested as O-demethylation of the quinazoline O-methyl group, which was observed in six out of eighteen tentative metabolites. Tentative metabolites M2, M8, M11, M12 and M13 with O-demethylation modification all had an intact morpholine ring. The results suggested that once O-demethylation of the methoxy group had occurred, the morpholine ring would keep intact. Therefore, tentative metabolite M9 was proposed to be metabolized by partial removal of the morpholine ring before O-demethylation. The O-demethylation reaction to the active tramadol metabolite was catalyzed by CYP2D6. The O-demethylation modification on gefitinib was considered to be catalyzed by CYP2D6 because CYP2D6 was demonstrated to catalyze O-demethylation reaction of tramadol metabolites with with chemical structure of methoxy group [21].

The substitution of the fluorine atom was the third major metabolic pathway for gefitinib in humans *in vivo*. The lipophilicity of molecules could be significantly increased if the C-F bond exists. In contrast, the hydrophilicity of gefitinib could be increased by replacing the fluorine atom with a hydroxyl group or sulfate group, leading to drug excretion. Additionally, the removal of a partial or complete 3-chloro-4-fluoroaniline-substituted group was observed in M3, M6 and M7. These tentative metabolites were not shown in a previous *in vitro* study based on human microsomes, which were newly discovered in human *in vivo* metabolism. The existing of the M3, M6 and M7 suggested that intestinal flora metabolism of gefitinib might be involved.

## Conclusion

In summary, gefitinib is extensively metabolized in humans *in vivo*, and the metabolic pathways are complex. Metabolism on the propoxy-morpholine ring side chain and O-methyl of gefitinib plays a critical role in the metabolism of gefitinib in humans *in vivo*, which was similar to that found during the metabolism of gefitinib in human microsomes [10, 11, 14]. In addition, eight novel metabolites, M1, M2 M3, M6, M7, M8, M11 and M18, were newly discovered in the presented study and indicated that novel metabolic pathways exist in humans *in vivo* and that intestinal flora metabolism might be involved [22]. This result is expected to facilitate a greater understanding of the metabolism of gefitinib and its potentially toxic products *in vivo*.

## Supporting information

S1 File (DOCX)

S1 Fig Metabolic pathways of gefitinib in rat, dog and human in the previous report. (TIF)
