# Supplementary material for: Tentative identification of gefitinib metabolites in non-small-cell lung cancer patient plasma using ultra-performance liquid chromatography coupled with triple quadrupole time-of-flight mass spectrometry
Source: PLoS One. 2020 Jul 23;15(7):e0236523. doi: 10.1371/journal.pone.0236523 (PMC7377447; doi:10.1371/journal.pone.0236523)
Supplement: S1 File — (DOCX) [file pone.0236523.s001.docx]

**Tentative identification of gefitinib metabolites in non-small-cell lung cancer patient plasma using ultra-performance liquid chromatography coupled with triple quadrupole time-of-flight mass spectrometry**

Supporting information

## Tentative identification of gefitinib metabolites

Fragmentation pathways were proposed by analyzing the MS/MS spectrum of the gefitinib reference standard. The product ions of gefitinib with *m/z* 100.0827, 128.1134, and 360.0343 were observed. The prominent product ions with *m/z* 128.1134 and 100.0827 were assigned to the propoxy-morpholine ring side chain and the propoxy-morpholine ring side chain with loss of two CH2 (-28), respectively. The results suggested that the carbon-carbon bond between C-21 and C-22 positions and the carbon-nitrogen bond between C-23 and N-24 positions will be easily broken under CID fragmentation. The remaining product ion with *m/z* 360.0343 assigned to the parent compound with a loss of morpholine ring proved the fragmentation pathways. As a result, product ions with *m/z* 128.1134 and 100.0827 would be absent in metabolites with modification on the propoxy-morpholine ring side chain. Product ion with *m/z* 360.0343 would be absent in metabolites with modification on the parent structure.

Tentative metabolite M1: The MS/MS spectrum of M1 (Fig 3A) showed prominent ions at *m/z* 120.0793, 166.0919, 190.0489, 208.0571 and 355.1222. The presence of product ions *m/z* of 120.0793, 166.0919, 190.0489 and 208.0571 were attributed to taurine conjugated on the morpholine ring of the side chain. The remaining product ion with *m/z* 355.1222 was assigned to the parent compound with a loss of taurine and a partial morpholine ring. Based on the accurate mass and MS/MS spectrum, M1 was tentatively identified as a compound with dechlorination and taurine conjugation of the morpholine ring.

Tentative metabolite M2: Because the MS/MS spectrum of M2 was not obtained, M2 was considered a compound by metabolizing gefitinib with defluorination at the F-19 position and demethylation at the O-30 position (-18-14 Da from the parent compound) based on the measured exact molecular mass and the most likely metabolic position of parent compound structure.

Tentative metabolite M3: The CID product ion spectrum of M3 (Fig 3B) showed product ions at *m/z* 118.0678, 146.0567 and 188.0710, which suggested addition of two oxygen atoms on the morpholine ring of the side chain, and dehydrogenation was assigned to the remaining portion of the compound. As a result, M3 was tentatively identified as gefitinib metabolized by removal of 3-chloro-4-fluoroaniline, addition of two atoms on the morpholine ring, and dehydrogenation.

Tentative metabolite M4: The MS/MS spectrum of M4 (Fig 3C) showed prominent ions at *m/z* 100.0763, 128.1065 and 445.1648, which indicated no metabolic change on the morpholine ring of the side chain. Sulfate conjugation occurred at the quinazoline skeleton or 3-chloroaniline substitutional group. With consideration of the metabolic pathway, M4 was tentatively identified as gefitinib metabolized by replacing a fluorine atom with a sulfate group.

Tentative metabolite M5: The MS/MS spectrum of M5 (Fig 3D) showed prominent ions at *m/z* 100.0751, 128.1062 and 341.0107. The presence of product ions *m/z* of 100.0751 and 128.1062, same as that of gefitinib, indicated that no metabolic change occurred on the morpholine ring side chain. The remaining product ion with *m/z* 341.0107 was assigned to M5 with loss of a morpholine ring and one water molecule. Because any carbon atom adjacent to a tertiary carbon was a quaternary carbon on the quinazoline skeleton, the hydroxylation probably occurred on the 3-chloroaniline substitutional group, where the hydroxyl group could be dehydrated with an adjacent hydrogen atom. With consideration of the metabolic pathway, M5 was tentatively identified as gefitinib metabolized by replacing a fluorine atom with a hydroxyl group.

Tentative metabolite M6: The MS/MS spectrum of M6 (Fig 3E) showed prominent ions at *m/z* 100.0752, 128.1060, 145.0505 and 245.1418, suggesting no metabolic change in the morpholine ring side chain. Based on the fragmentation pattern, M6 was tentatively identified as gefitinib metabolized by removal of 3-chloro-4-fluoroaniline.

Tentative metabolite M7: The prominent ions of *m/z* 100.0749, 128.1060 and 260.0914 were observed in the MS/MS spectrum of M6 (Fig 3F). The presence of product ions *m/z* of 100.0749 and 128.1060, same as that of gefitinib, indicated that no metabolic change occurred on the morpholine ring side chain. The remaining product ion with *m/z* 260.0914 was assigned to M7 with loss of methoxyethane of the morpholine ring. Therefore, M7 was tentatively identified as gefitinib metabolized by removal of the 1-chloro-2-fluorobenzene group.

Tentative metabolite M8: The MS/MS spectrum of M8 (Fig 3G) showed prominent ions at *m/z* 128.1063, 346.0773 and 433.1424. The presence of product ion *m/z* of 128.1063, same as that of gefitinib, was attributed to the morpholine ring side chain. The product ion with *m/z* 346.0773 was assigned to M8 with simultaneous loss of glucuronide and a morpholine ring. The remaining product ion with *m/z* 433.1424 was assigned to M8 with loss of glucuronide. Based on the fragmentation pattern and metabolic pathways, M8 was tentatively identified as gefitinib metabolized by replacing O-methyl with glucuronide.

Tentative metabolite M9: The product ions of *m/z* 74.0612, 102.0920, 306.0427 and 346.0739 were observed in the MS/MS spectrum of M9 (Fig 3H). The presence of product ions of *m/z* 74.0612 and 102.0920 were approximately 26 Da less than those of the fragment of the gefitinib propoxy-morpholine ring side chain 100.0827 and 128.1134, which indicated that double demethylation and hydrogenation occurred on the propoxy-morpholine ring side chain. The product ions with *m/z* 306.0427 and 346.0739 were assigned to M9 with O-methylation. As a result, M9 was tentatively identified as gefitinib metabolized by demethylation at the O-30 position and double demethylation on the opening morpholine ring.

Tentative metabolite M10: The MS/MS spectrum of M10 (Fig 3I) showed prominent ions at *m/z* 74.0721, 102.0941, 305.0355 and 320.0600. The presence of product ions *m/z* of 74.0721 and 102.0941 was the same as that of M9, which indicated that double demethylation and hydrogenation occurred on the propoxy-morpholine ring side chain. The molecular weight of M10 was approximately 14 Da more than that of M9, suggesting the existence of O-methylation. As a result, M10 was tentatively identified as M9 without O-demethylation.

Tentative metabolite M11: The MS/MS spectrum of M11 (Fig 3J) showed prominent ions at *m/z* 100.0751, 128.1069, 306.0297, 320.0404 and 348.0724. The presence of product ions *m/z* of 100.0751 and 128.1069, same as that of gefitinib, indicated that no metabolic change occurred on the propoxy-morpholine ring side chain. Therefore, the demethylation reaction was proposed to occur at the O-30 position. The presence of product ions *m/z* of 306.0297, 320.0404 and 348.0724 was assigned to M11 with loss of a sequence of portions of the side chain. As a result, M11 was tentatively identified as gefitinib metabolized by O-demethylation and hydrogenation on the quinazoline skeleton or the N-[1-chloro-2-fluorobenzene] substituent group.

Tentative metabolite M12: As shown in Fig 3K, the prominent ions of *m/z* 100.0835, 128.1168 and 346.0717 were observed on the MS/MS spectrum. The presence of product ions *m/z* of 100.0835 and 128.1168, same as that of gefitinib, indicated that no metabolic change occurred on the propoxy-morpholine ring side chain. The remaining product ion with *m/z* 346.0717 was assigned to M12 with loss of a morpholine ring. Considering the metabolic pathways, M12 was tentatively identified as O-desmethyl gefitinib.

Tentative metabolite M13: The MS/MS spectrum of M13 (Fig 3L) showed prominent ions at *m/z* 74.1010, 128.1167 and 376.2627. The presence of product ions *m/z* of 74.1010 and 128.1167 was assigned to the propoxy-morpholine ring side chain, same as the parent drug. The demethylation was suggested as O-demethylation. The oxidation was assigned to the quinazoline skeleton or N-[1-chloro-2-fluorobenzene] substituent group based on the fragmentation pattern and metabolic pathway. Therefore, M13 was tentatively identified as hydroxylation of O-desmethyl gefitinib.

Tentative metabolite M14: The prominent ions of *m/z* 86.0609, 142.0884 and 318.0652 were observed on the MS/MS spectrum (Fig 3M). The presence of product ions *m/z* of 86.0609 and 142.0884 was attributed to the oxidative morpholine ring side chain with dehydrogenation. The product ion of *m/z* 318.0652 was assigned to M14 with loss of a O-propoxy-morpholine group. Based on the metabolic pathway, defluorination and oxidation were considered as replacement of a fluorine atom with a hydroxyl group. As a result, M14 was tentatively identified as gefitinib metabolized by oxidation and dehydrogenation to the morpholine ring and a hydroxyl group substituted to the fluorine atom.

Tentative metabolite M15: The MS/MS spectrum of M15 (Fig 3N) showed prominent ions at *m/z* 158.0815, 304.0294 and 320.0618. The presence of a product ion *m/z* of 158.0815 was approximately 30 Da greater than the fragment of the propoxy-morpholine ring side chain of gefitinib at *m/z* 128.1134 and 16 Da greater than the fragment of the oxidative propoxy-morpholine ring side chain with dehydrogenation of M14 at *m/z* 142.0884, which indicated that double hydroxylation and dehydrogenation occurred on the propoxy-morpholine ring side chain of M15. The presence of product ions *m/z* of 304.0294 and 320.0618 was assigned to M15 with loss of a propoxy-morpholine ring side chain. Therefore, M15 was tentatively identified as M14 metabolized by further oxidation on the propoxy-morpholine ring side chain.

Tentative metabolite M16: Because the MS/MS spectrum of M16 was not obtained, M16 was tentatively proposed to be gefitinib metabolized by loss of morpholine, hydroxylation and carbonylation at the C-23 position referenced to the presented study.

Tentative metabolite M17: As shown in Fig 3O, product ions with *m/z* of 86.0610, 114.0554, 142.0863 and 320.0596 were observed. The presence of product ions *m/z* of 86.0610, 114.0554 and 142.0863, similar to those of M14, was attributed to the oxidative propoxy-morpholine ring side chain with dehydrogenation. The product ion of *m/z* 320.0596 was assigned to M17 with loss of a O-propoxy-morpholine group. As a result, M17 was tentatively identified as gefitinib metabolized by oxidation and dehydrogenation to the morpholine ring.

Tentative metabolite M18: As shown in Fig 3O and 3P, M17 and M18 had three pairs of identical product ions with m/z of 86.0610 (86.0595), 114.0554 (114.0551) and 142.0863 (142.0861), which indicated the same propoxy-morpholine ring side chain. Compared with the product ions of gefitinib assigned to the side chain, an equal difference of 14 Da was observed, which suggested the metabolic change in oxidation and dehydrogenation on the propoxy-morpholine ring side chain. The remaining product ions of M17 and M18 with m/z 320.0596 and 322.0570 were assigned to their compounds with loss of the propoxy-morpholine ring side chain, respectively. The difference between the two product ions of 2 Da indicated that the addition of two hydrogen atoms of M18 occurred on the quinazoline skeleton or the 3-chloro-4-fluoroaniline group (Fig 3P). Considering the metabolic pathways, M18 was identified as hydrogenated M17.
