# Supplementary figures and images for: Tentative identification of gefitinib metabolites in non-small-cell lung cancer patient plasma using ultra-performance liquid chromatography coupled with triple quadrupole time-of-flight mass spectrometry
Source: PLoS One. 2020 Jul 23;15(7):e0236523. doi: 10.1371/journal.pone.0236523 (PMC7377447; doi:10.1371/journal.pone.0236523)

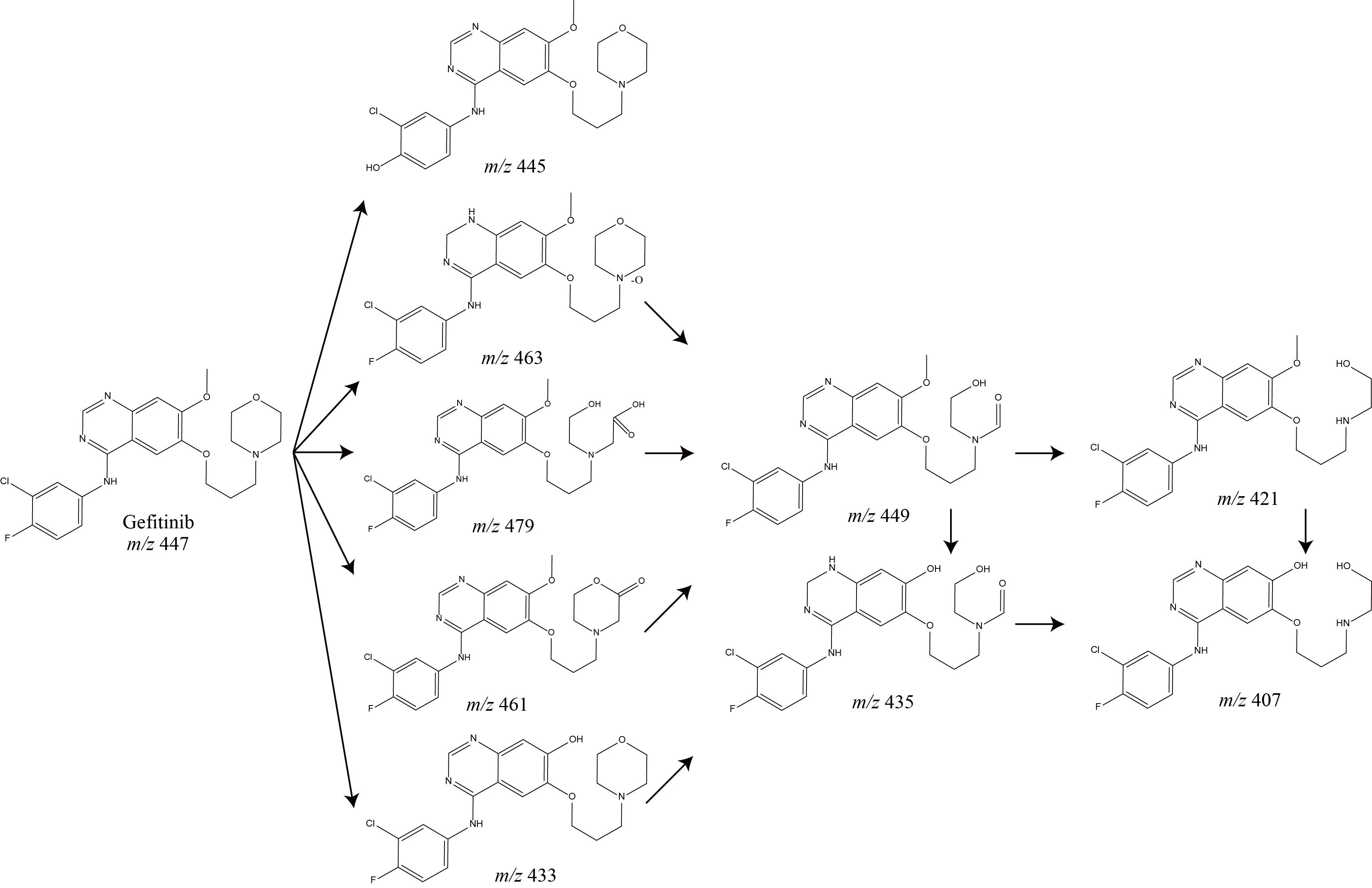

Supplement: S1 Fig — (TIF) [file pone.0236523.s002.tif]
